# Fistule bilio-pleurale post traumatique: à propos d’un cas

**DOI:** 10.4314/pamj.v4i1.53596

**Published:** 2010-03-24

**Authors:** Mohamed Smahi, Marouane Lakranbi, Nada Tazi, Khalid Maazaz

**Affiliations:** 1 Service de chirurgie thoracique, CHU Hassan II, Fès, Maroc;; 2 Service de chirurgie viscérale B, CHU Hassan II, Fès, Maroc

**Keywords:** Fistule bilio pleurale, plaie thoraco abdominale, Chirurgie, Pleuro biliary fistulae, Thoraco abdominal injury, Surgery

## Abstract

Les fistules bilio thoraciques post traumatiques sont extrêmement rares, leur traitement peut être conservateur mettant en œuvre des techniques de radiologie et d’endoscopie interventionnelle ou chirurgical si échec du premier. Nous rapportons une observation de fistule bilio pleurale post traumatique où l’indication du traitement chirurgical de première intention a été indiscutable vu le sepsis et l’instabilité hémodynamique.

## Introduction

Les fistules biliothoraciques qu’elles soient bilio pleurales et/ou bilio bronchiques sont le plus souvent d’origine hydatique ou congénitale, rarement iatrogène ou post opératoire après une chirurgie hépato-biliaire et exceptionnellement post traumatique après un traumatisme pénétrant thoraco-abdominal [1]. Si l’attitude chirurgicale agressive de première intention qui donnait d’excellents résultats [2] a été récemment remise en question vu les progrès réalisés dans les domaines de la radiologie et l’endoscopie interventionnelles [2], son indication reste indiscutable en cas de sepsis avec instabilité hémodynamique.

Nous rapportons un cas de fistule bilio-pleurale post traumatique à travers lequel nous décrivons les aspects épidémiologiques, cliniques, radiologiques et thérapeutiques d’une telle rare entité.

## Patient et observation

**Figure 1:**
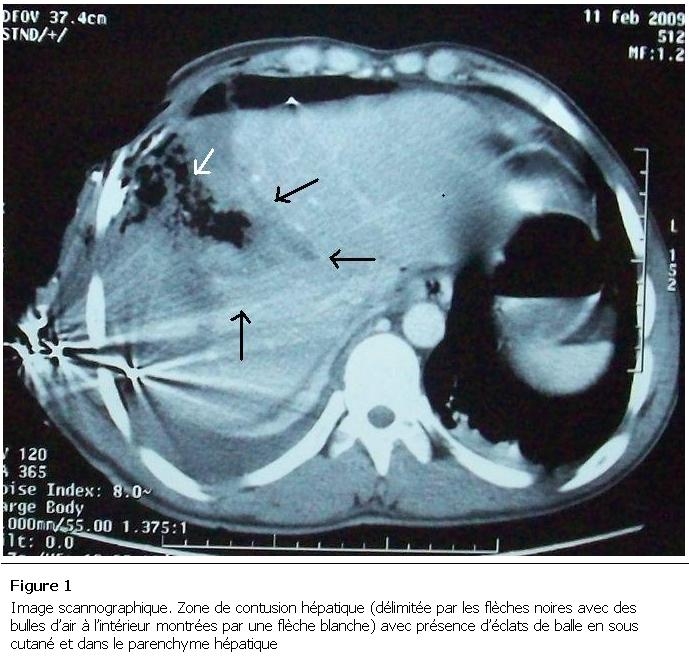
Image scannographique. Zone de contusion hépatique (délimitée par les flèches noires avec des bulles d’air à l’intérieur montrées par une flèche blanche) avec présence d’éclats de balle en sous cutané et dans le parenchyme hépatique

**Figure 2:**
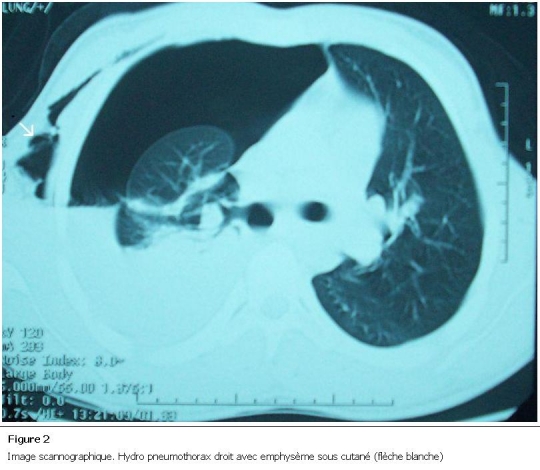
Image scannographique. Hydro pneumothorax droit avec emphysème sous cutané (flèche blanche)

**Figure 3:**
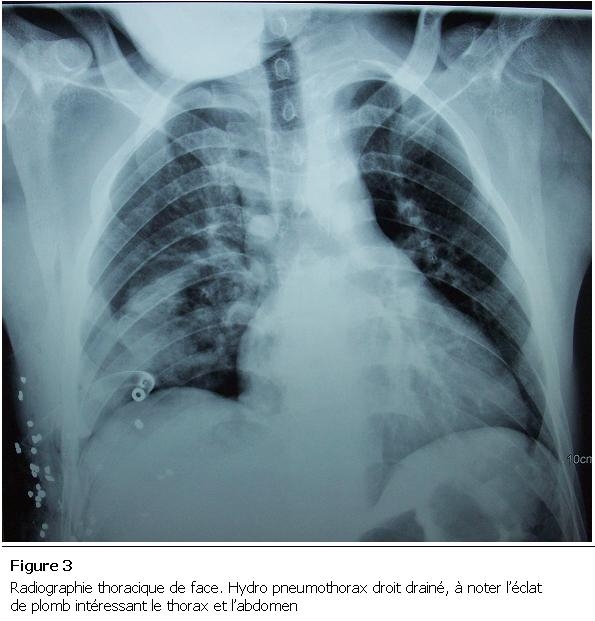
Radiographie thoracique de face. Hydro pneumothorax droit drainé, à noter l’éclat de plomb intéressant le thorax et l’abdomen

Monsieur K. A, âgé de 20 ans, admis aux urgences pour dyspnée avec sepsis suite à un traumatisme pénétrant thoraco-abdominal par éclats de balle accidentel, avec hémo-pneumothorax droit drainé sept jours avant. L’examen clinique à l’admission trouvait un patient polypneique, fébrile à 39° C, un pouls à 114 battements/min et une tension artérielle de 90/60 mmHg. A l’inspection on a noté la présence de deux cicatrices de l’hémi thorax droit dont l’une correspondait à la plaie thoracique suturée et à l’autre à l’orifice de drainage thoracique. On a noté une défense de l’hypochondre droit et des signes d’épanchement pleural liquidien droits consécutivement à l’examen abdominal et pleuro pulmonaire. La radiographie thoracique objectivait les éclats de plomb avec un épanchement pleural mixte. La tomodensitométrie thoraco-abdominale a mis en évidence une contusion hépatique (Figure 1) avec collection inter hépato diaphragmatique, associée à un hydro pneumothorax droit (Figure 2). Un drainage thoracique a été effectué (Figure 3), il a ramené un litre de bile, stérile à l’examen bactériologique.

Le bilan biologique a permis de noter une hyperleucocytose à 21300/mm^3^, une hémoglobine à 8.5g/dl ayant nécessité une transfusion sanguine avec une CRP élevée.

**Figure 4:**
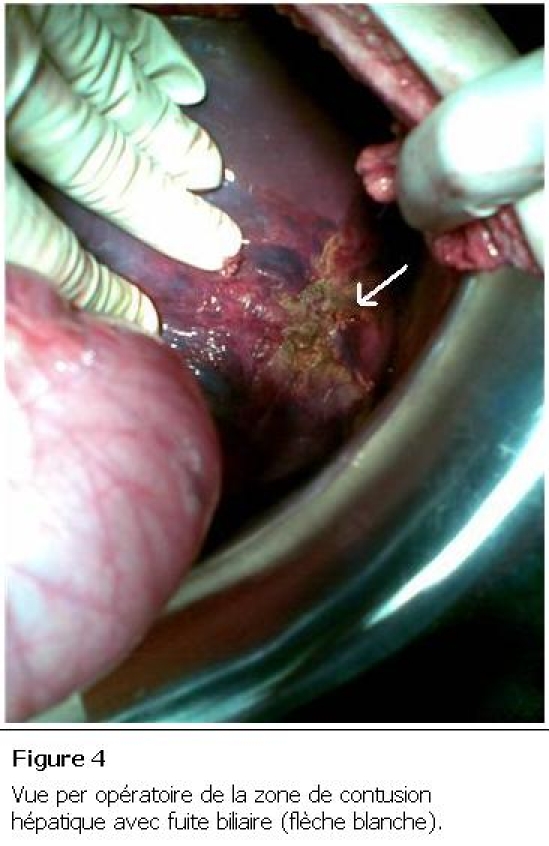
Vue per opératoire de la zone de contusion hépatique avec fuite biliaire (flèche blanche)

**Figure 5:**
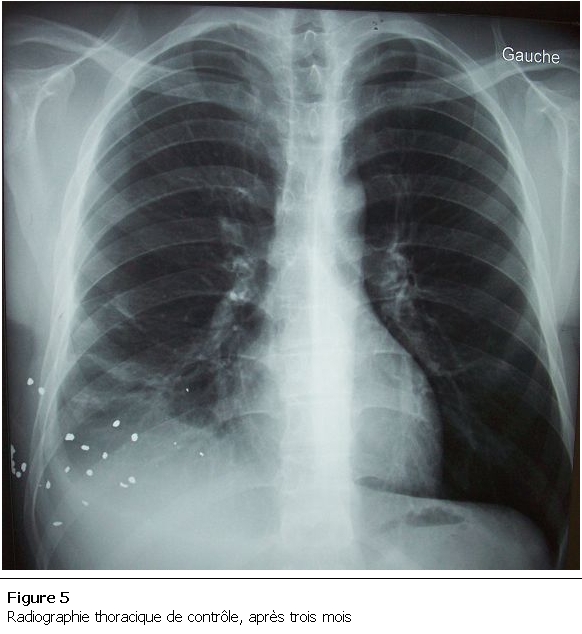
Radiographie thoracique de contrôle, après trois mois

Le patient a été opéré, une laparotomie médiane a été réalisée, mettant en évidence une zone de contusion du dôme hépatique (Figure 4) avec collection biliaire en inter hépato phrénique, communiquant avec la plèvre droite par un trajet fistuleux. Le geste chirurgical a consisté en une déconnexion hépato diaphragmatique, mise à plat de la collection biliaire inter hépato diaphragmatique et du trajet fistuleux, avec drainage externe de la collection par des lames de DELBET, vu la fuite biliaire importante et l’impossibilité de l’aveuglement des fistules biliaires au niveau de la zone contuse et phrénoplastie. En post opératoire, le drainage pleural et abdominal ramenait 200 ml de bile quotidiennement, puis s’est tari progressivement. Le patient est déclaré sortant après une hospitalisation de 21 jours. Le patient revu trois mois après en consultation, le contrôle clinique et radiologique (Figure 5) étaient satisfaisants.

## Discussion

L’incidence exacte des fistules bilio-thoraciques (FBT) post traumatiques n’est pas connue [2], des cas sporadiques sont rapportés dans la littérature. Navsaria [2] a recensé en 2002, 34 cas rapportés dans la littérature anglaise depuis 1994, et seulement 9 cas depuis 1999. Cette complication survient dans 2 à 4% des traumatismes hépatiques [3] quelque soit le mécanisme. Pour nous, habitués à la chirurgie des fistules bilio-bronchiques et/ou bilio-pleurales d’origine hydatique vu notre contexte endémique, c’est le premier cas de fistule bilio-pleurale post traumatique.

Leur mécanisme de survenue peut être expliqué lors d’un traumatisme pénétrant thoraco-abdominal, par la survenue d’une plaie hépatique avec fuite biliaire et la constitution d’une collection biliaire sous phrénique, qui du fait de son effet corrosif, son infection et la pression pleurale négative finit par se fistuliser dans l’arbre bronchique et/ou la cavité pleurale.

Les fistules bilio-thoraciques peuvent se manifester cliniquement par une biliptysie, un épanchement pleural bilieux, une fuite biliaire à travers une fistule cutanée, associés ou non à une collection sous phrénique ou une péritonite biliaire. Un état fébrile avec une hyperleucocytose sont observés dans 50% des cas [3], comme c’est le cas ici. L’ictère n’est pas observé tant qu’il n’y a d’obstruction des voies biliaires.

Le bilan radiologique repose sur la radiographie thoracique, l’échographie abdominale qui permet une bonne étude des voies biliaires intra et surtout extra-hépatiques à la recherche d’un éventuel obstacle, mais surtout sur la tomodensitométrie thoraco-abdominale injectée, qui permet une meilleure analyse des parenchymes pulmonaire et hépatique, la recherche de collection pleurale et abdominale notamment sous phrénique, la recherche d’une discontinuité diaphragmatique qui est souvent difficile à mettre en évidence.

Ces examens peuvent être complétés par la cholangio-pancréatographie endoscopique rétrograde ou la cholangiographie percutanée transhépatique permettant l’opacification et la mise en évidence de la fistule biliaire.

Le traitement classiquement exclusivement chirurgical, est calqué sur celui des fistules bilio-bronchiques notamment d’origine hydatique. La voie d’abord est souvent une thoracotomie basse si les dégâts pleuro pulmonaires sont importants, parfois une laparotomie seule suffit comme c’est le cas ici. Il permet la résection des foyers pulmonaires détruits, la décortication pleuro pulmonaire, la déconnexion hépato diaphragmatique, ainsi que le drainage des collections hépatiques, sous phréniques et la phrénoplastie [1].

Ce traitement chirurgical, est de plus en plus supplée par un traitement médical conservateur, basé sur l’antibiothérapie adaptée, le drainage percutané des collections abdominales parfois pleurales, et en l’absence d’amélioration dans les 72 à 96 heures qui suivent, une sphinctérotomie endoscopique est indiquée pour favoriser le tarissement de la fuite biliaire [5]. Ce traitement a pour inconvénients une logue durée d’hospitalisation et des manipulations radiologiques multiples.

Actuellement il n’y a pas de consensus concernant le traitement à adopter, mais des recommandations semblent se dégager privilégiant le traitement conservateur en premier, à condition qu’il n’entraine pas de morbidité supplémentaire en retardant une chirurgie secondaire s’elle est indiquée. Ce traitement conservateur permet la guérison dans 60% des cas [6]. La chirurgie est indiqué en cas de FBT avec sepsis et instabilité hémodynamique ou de lésions bilio pleuro pulmonaires complexes associées.

## Conclusion

La fistule bilio pleurale et/ou bilio-bronchique post traumatique est une complication rare et grave, entrainant une déperdition biliaire avec des conséquences hydro électrolytiques, septiques et respiratoires, dont le traitement est de plus en plus conservateur, mais la chirurgie garde sa place et doit être pratiquées sans délai s’elle est indiquée.

## Conflits d’intérêts

Les auteurs ne déclarent aucuns conflits d’intérêts.

## Contribution des auteurs

Prise en charge médico-chirurgicale du patient : **MS**, **ML NT** et **KM**. Rédaction du manuscrit : **MS** et **ML**.
